# The Italian Score for Organ Allocation: A Ten-Year Monocentric Retrospective Analysis in Liver Transplantation for Hepatocellular Carcinoma

**DOI:** 10.3390/cancers17101720

**Published:** 2025-05-21

**Authors:** Enrico Prosperi, Matteo Cescon, Quirino Lai, Chiara Bonatti, Edoardo Prosperi, Francesca Rizzo, Lorenzo Maroni, Andrea Laurenzi, Matteo Serenari, Maria Cristina Morelli, Matteo Ravaioli

**Affiliations:** 1 Hepatobiliary and Transplant Surgery Unit, IRCCS Azienda Ospedaliero-Universitaria di Bologna, 40138 Bologna, Italy; matteo.cescon@unibo.it (M.C.); chiara.bonatti3@unibo.it (C.B.); edoardo.prosperi@studio.unibo.it (E.P.); francesca.rizzo2505@gmail.com (F.R.); lorenzo.maroni@aosp.bo.it (L.M.); andrea.laurenzi@aosp.bo.it (A.L.); matteo.serenari@gmail.com (M.S.); 2 Department of Medical and Surgical Sciences (DIMEC), University of Bologna, 40126 Bologna, Italy; 3 General Surgery and Organ Transplantation Unit, AOU Policlinico Umberto I, Sapienza University of Rome, 00185 Rome, Italy; quirino.lai@uniroma1.it; 4 Internal Medicine Unit for the Treatment of Severe Organ Failure, IRCCS Azienda Ospedaliero-Universitaria di Bologna, 40138 Bologna, Italy; mariacristina.morelli@aosp.bo.it

**Keywords:** liver transplantation, liver allocation, hepatocellular carcinoma, locoregional treatment, prioritization, ethical allocation, intention to treat survival, competing risk regression

## Abstract

Background: The Italian Score for Organ Allocation (ISO), a transplant benefit oriented allocation system, was introduced in Italy in 2016. The main objective of this study is to identify risk factors for Drop-Out in hepatocellular (HCC) patients enlisted for LT before (Pre-ISO Era) and after ISO (ISO Era) introduction, while the secondary objective is to evaluate the survival results. Methods: CIFs for liver transplantation and Drop-Out were estimated and compared between eras. Factors associated with Drop-Out were identified through multivariable competing risks regression. Survival results were compared using the log-rank test. Results: Between 2011 and 2020, 410 patients with HCC were listed for LT. We observed 103 vs. 217 LT and 49 vs. 41 Drop-Outs (*p* < 0.001) during the Pre-ISO and ISO Era, respectively. In the multivariable analysis, ISO ([sHR] 0.43; 95%CI 0.28–0.66, *p* < 0.001) and Alcoholic Cirrhosis ([sHR] 0.27, 95%CI 0.11–0.70; *p* = 0.007) were revealed to be protective factors for Drop-Out. One year after listing, the CI for Drop-Out decreased from 13.2% to 6.2% (*p* = 0.02). Despite no differences observed in post-LT survival, a significant difference in the intention-to-treat survival from enlisting was found (*p* = 0.0019). Conclusions: Among other factors, ISO results were protective for the Drop-Out risk in HCC patients awaiting LT, with a benefit in ITT-OS survival.

## Simple Summary

Liver transplantation is the most effective treatment to improve survival in patients with hepatocellular carcinoma. However, the limited availability of donor organs makes fair and effective allocation essential. In 2016, Italy introduced the Italian Score for Organ Allocation, a system based on transplant benefit principles, to better prioritize patients at highest risk of being removed from the waiting list. This study evaluates the impact of the Italian Score for Organ Allocation over the past decade, focusing on changes in drop-out rates from the waiting list and post-transplant survival.

## 1. Introduction

Liver transplantation (LT) is the most effective therapy for early-to-intermediate stages of hepatocellular carcinoma (HCC). However, due to organ shortage, the use of this scarce medical resource is limited, and multiple theoretical bases are driving the search for the strategies to allocate liver grafts. At present, most allocation policies worldwide rely on the model for end-stage liver disease (MELD) score [1], but it is well described that the MELD score does not always capture the gravity of the patients waiting for an organ, particularly for patients with HCC [2]. In this sense, an ideal allocation policy would prioritize the patients with a higher risk of Drop-Out from the waiting list but simultaneously limit the risk of HCC recurrence. Different types of allocation have been applied over the years, following national and regional policies. Since 2016, a new allocation score, the Italian Score for Organ Allocation (ISO), has been introduced in Italy [3]. The new score relies on the MELD score for non-HCC patients, with different additional points related to the severity of the underlying liver disease (ULD) or the complications of portal hypertension. In HCC patients, the ISO relies on the behavior of HCC regarding the pathologic response to locoregional therapies (LRTs) and, consequently, the Drop-Out risk. In this setting, patients with HCC are prioritized in the presence of recurrence or residual disease after LRT, with higher priority for early (<12 months) recurrence and lower priority for late recurrence (>24 months). Conversely, a tumor with a post-LRT sustained completed response does not receive additional priority.

The locoregional ablation application strategy depends on the Barcelona Clinic Liver Cancer [4] algorithm. For BCLC-A cases, the possible LRTs are Liver Resection (LR) or ablative therapies (radiofrequency ablation (RFA) [5], microwave ablation (MWA) [6], and percutaneous ethanol injection (PEI) [7]), while for BCLC-B patients, intra-arterial therapies like trans-arterial chemoembolization (TACE) [8] or Yttrium-90 Radioembolization (Y-90) [9] are used. In a limited number of patients, new treatments such as systemic therapy and stereotactic radiotherapy are used, mostly in combination with standard locoregional therapies.

The primary outcome of this study is to compare the Drop-Out rate among HCC patients listed for LT during the Pre-ISO and ISO Eras. Secondary outcomes include comparisons of post-LT recurrence-free survival (RFS), overall survival (OS), and intention-to-treat overall survival (ITT-OS) from the time of listing.

## 2. Patients and Methods

### 2.1. Study Design

The Liver Transplant Unit of Bologna has consistently ranked among the top five most active liver transplant centers in Italy over the past years, with patient volume and listing policies that are representative of national practice [10]. This is a retrospective monocentric study involving all adult (≥18 years) patients diagnosed with HCC listed for LT from 2011 to 2020 at the University of Bologna Liver Transplantation Unit. All the patients receiving a living-donor LT (n = 4) or with an incidental diagnosis of HCC at the explant pathology (n = 5) were excluded. A total of 410 patients were enrolled in the study. The entire cohort was stratified into two groups according to the application of the ISO in January 2016: Pre-ISO (2011–2015; n = 152) and ISO Era (2016–2020; n = 258) (Supplementary Figure S1). The indications for LT for HCC at the University of Bologna have been described elsewhere [11,12].

**Figure 1:**
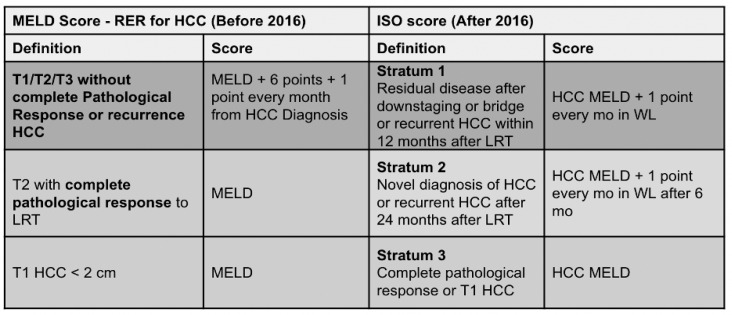
Allocation policy. Abbreviations: HCC, hepatocellular carcinoma; ISO, Italian Score for Organ Allocation; MELD, model for end-stage liver disease.

As previously stated, a new allocation system—the ISO—was introduced in 2016, marking a substantial change from the previous model. In the Pre-ISO Era, the allocation rule for HCC followed the MELD Score Regione Emilia-Romagna (RER), named after the Italian region where the transplant center is located. (Figure 1). With this allocation system, no additional points were assigned for patients with a single HCC tumor inferior to 2 cm (T1) [13] or T2 HCC within Milan Criteria with complete pathological response to LRT. Conversely, for patients with up to three HCC tumors between 2 and 3 cm or with a single HCC tumor between 3.1 and 5 cm with partial response after LRT or with evidence of disease progression, 6 points were initially added, with an additional point added each month, starting from the date of HCC diagnosis, with the possibility of class modification.

Since 2016, HCC patients have been divided into three risk classes adjusted according to the clinical, radiological, and AFP variations after LRT [11]. The classes are as follows: Stratum 1 = residual disease after downstaging or bridge, or recurrence of HCC within 12 months after LRT; Stratum 2 = novel diagnosis of HCC, or recurrence of HCC after 24 months after LRT; Stratum 3 = complete response to LRT, or T1 HCC. The Stratum Score is calculated by adding to the HCC-MELD [14] 1 point for each month since insertion in the waiting list (WL) for Stratum 1, 1 point each month after 6 months since insertion in the WL for Stratum 2, or HCC-MELD without additional points for Stratum 3. In this system, a patient could move from one class to another, having the opportunity to dynamically modify their position in the WL according to the post-LRT response. Response to LRT was defined according to the Response Evaluation Criteria in Solid Tumors (RECIST) [15].

### 2.2. Study Endpoints

The primary outcome of this study is to assess the risk of Drop-Out before and after ISO adoption using a competing risks analysis and identify risk factors for Drop-Out. Secondary outcomes include evaluating differences in recurrence-free survival (RFS), overall survival (OS), and intention-to-treat overall survival (ITT-OS) between the two periods.

### 2.3. Ethics

Local institutional review boards of the University of Bologna approved this study.

### 2.4. Data Collection

This retrospective single-center study utilized data derived from a prospectively maintained local registry, including variables such as age, sex, Child–Pugh score, and laboratory MELD score. The MELD score at the exit was the last MELD score before LT, or the last observed MELD score reported for patients excluded from the WL. The other variables collected included ULD, previous abdominal surgical operation, the presence and extension of portal vein thrombosis (PVT), and BMI. HCC variables included the number of nodules, the diameter of the biggest lesion, and AFP level (ng/mL) evaluated at diagnosis and the last radiological imaging before LT. Post-LT pathological data were also collected, including the number and size of HCC, tumor grading, and microvascular Invasion (mVI) presence. The time in the list was calculated as the difference between the WL entry and exit dates. The WL time was expressed in months. In accordance with prior research, Drop-Out from the waiting list—whether due to death, clinical deterioration, or tumor progression—was classified under a single outcome category (‘Drop-Out’), as these events commonly reflect terminal disease conditions in patients with HCC [16]. Complete pathological response was defined as the absence of active HCC at the last imaging pre-LT (Contrast-Enhanced Computed Tomography or Magnetic Resonance). RFS was calculated as the difference between the date of LT and recurrence of HCC after LT. OS was calculated as the difference between the date of LT and death or last follow-up. ITT-OS was calculated as the difference between the date of WL inscription and Drop-Out, death, or last follow-up. The enrollment of the patients was stopped on 31 December 2020. Every transplanted patient has a minimum follow-up of two years to detect the appearance of recurrence of HCC [17], and the median RFS of the transplanted cohort in months is 52 (IQR 28–72).

### 2.5. Follow-Up Period

Patients were followed up from the waiting list entry date and censored at the occurrence of Drop-Out or LT. Patients receiving LT were followed from the day of LT to the date of rHCC for calculation of RFS and death or last follow-up for OS. The ITT survival was considered the difference between the Drop-Out date and WL registration for Drop-Out patients and the difference between death or the last FU for LT patients.

### 2.6. Statistical Analysis

Patient characteristics were summarized using medians and interquartile ranges (IQRs) for continuous variables and proportions for categorical variables. Comparisons of continuous variables were performed using the Mann–Whitney U test, while categorical variables were compared using Pearson’s chi-square test or Fisher’s exact test, as appropriate.

A competing risks regression model was applied to estimate sub-distribution hazard ratios (sHRs) and 95% confidence intervals (CIs) for the outcomes “Drop-Out” and “Liver Transplantation”, considering “Liver Transplantation” and “Drop-Out” as the respective competing events.

To identify risk factors associated with Drop-Out within 3 years of waitlist (WL) inscription, a multivariable logistic regression model was developed using a stepwise approach, starting from pairwise comparisons with *p*-values < 0.1. Sub-hazard ratios (sHRs) and 95%CIs were reported. To assess the proportional hazards assumption in the competing risks regression models, we tested for time-varying effects by including interaction terms between covariates and the log of time. Specifically, we generated interaction variables between each covariate and the natural logarithm of waiting list time and included them in the Fine and Gray sub-distribution hazard models. Significant interactions were interpreted as evidence of violation of the proportional hazards assumption.

The cumulative incidence functions for WL Drop-Out and liver transplantation were compared using the method described by Pepe and Mori, implemented via the user-written Stata command stpepemori. The survival results were compared with the Log Rank test. A *p*-value < 0.05 was considered statistically significant. All statistical analyses were performed using Stata versions 17–18 (StataCorp, College Station, TX, USA).

## 3. Results

### 3.1. Listing Characteristics

Between 1 January 2011 and 31 December 2020, 410 patients with HCC on the liver transplant waiting list either underwent liver transplantation or dropped out.

The general characteristics of the entire cohort at enlisting are summarized in Table 1. The median age at waiting list (WL) inscription was 57.4 years (IQR = 51.8–62.6), with 83.9% of the patients being males and the WL duration being 10.6 months (IQR = 4.4–18.1). In the Pre-ISO Era, the patients were enlisted with a higher MELD score (*p* < 0.001) and Child–Pugh Score (*p* = 0.007). There were no differences between the two groups regarding sex, blood group, ULD, and portal vein thrombosis (PVT).

HCC characteristics at enlisting and LRT are shown in Table 2. The groups shared similarities in the number of nodules (*p* = 0.058), the diameter of the major lesion (*p* = 0.405), and AFP values at diagnosis. In the entire cohort, more than 90% of the patients underwent at least one neo-adjuvant treatment before LT; no relevant differences in the application of LRT were found. In the ISO Era, 14 patients (7.3%) received other treatments prior to LT, including Sorafenib (n = 8) [18], stereotactic radiotherapy (n = 5) [19], and Capecitabine plus Cyclophosphamide (n = 1), compared to 1 (1%) patient receiving Sorafenib in the Pre-ISO Era.

Ninety patients (21.9%) dropped out in the entire cohort. A total of 49/152 (32.2%) vs. 41/258 (15.9%) Drop-Out cases were reported in the Pre-ISO and ISO groups, respectively (*p* < 0.001).

The leading cause of Drop-Out was worsening of clinical conditions (48/410; 11.7%), followed by death (42/410; 10.2%) (*p* < 0.001). After splitting these two causes in the two groups, more cases of death and clinical worsening in patients on the WL were reported in the Pre-ISO group. More specifically, 22/152 (14.5%) vs. 20/258 (7.8%) deaths and 27/152 (17.8%) vs. 21/258 (8.1%) clinical worsening cases were reported (*p* < 0.001).

The median waiting time from waiting list inscription to either Drop-Out or liver transplantation did not differ between the two periods (11 vs. 10 months; *p* = 0.518).

### 3.2. Risk Factors for Waiting List Drop-Out

**Figure 2:**
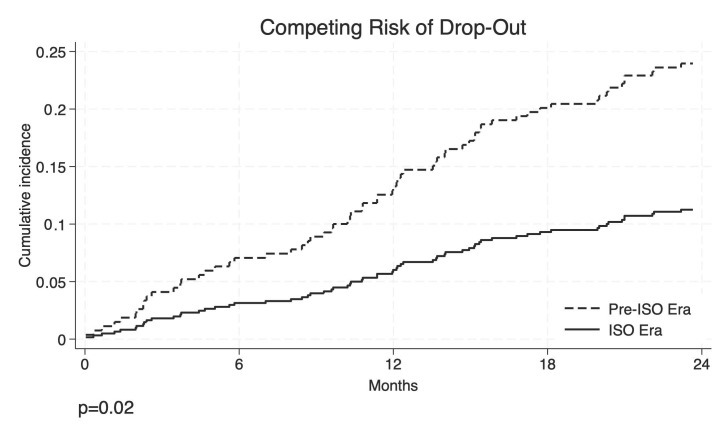
Cumulative incidence of Drop-Out.

Comparing the different cumulative incidences in the Pre-ISO vs. ISO period, values of 4.0% (95%CI = 2.0–8.8) vs. 1.6% (95%CI = 0.5–3.7) at 3 months, 8.6% (95%CI = 4.8–13.7) vs. 2.3% (95%CI = 1.0–4.8) at 6 months, and 13.2% (95%CI = 8.4–19.1) vs. 6.2% (95%CI = 3.7–9.6) at 12 months were reported, respectively. Pepe and Mori’s test showed a lower cumulative incidence of Drop-Out in the ISO period compared to the Pre-ISO period (*p* = 0.02) (Figure 2).

**Figure 3:**
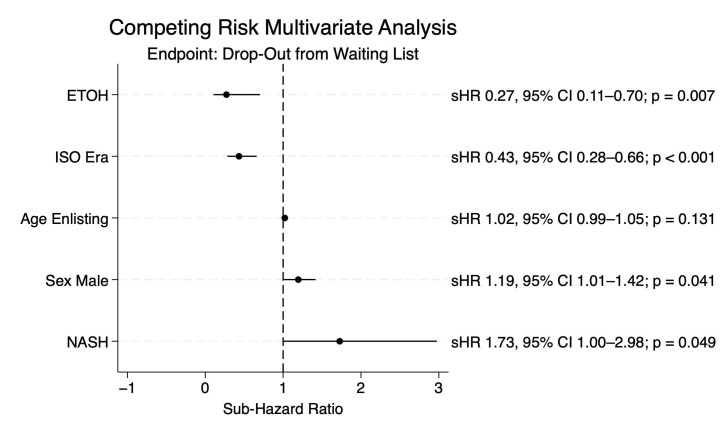
Competing risks multivariable analysis. Abbreviations: CI, confidence interval; HCV, hepatitis C virus; ISO, Italian Score for Organ Allocation; NASH, nonalcoholic steato-hepatitis; PVT, portal vein thrombosis; sHR, sub-hazard ratio. Note: Sex was modeled as a time-varying covariate (interaction with log-transformed time).

In the multivariable competing risks analysis (Figure 3), considering liver transplantation (LT) as a competing event for Drop-Out from the waiting list (WL), listing during the ISO era was associated with a significantly lower sub-hazard ratio of Drop-Out (sHR 0.43, 95%CI 0.28–0.66; *p* < 0.001). Alcohol-related etiology also showed a significantly reduced risk (sHR 0.27, 95%CI 0.11–0.70; *p* = 0.007). NASH was associated with a higher sub-hazard ratio of Drop-Out (sHR 1.73, 95%CI 1.00–2.98; *p* = 0.049). Age at listing (sHR 1.02, 95%CI 0.99–1.05; *p* = 0.131) did not reach statistical significance. Sex was modeled as a time-varying covariate, and its effect increased over time. The interaction term was statistically significant (sHR 1.19, 95%CI 1.01–1.42; *p* = 0.041), indicating that the risk associated with being male increased as follow-up time progressed.

### 3.3. Liver Transplantation Characteristics

In the study period, 320 patients underwent LT. More specifically, 103/152 (67.8%) vs. 217/258 (84.1%) were transplanted in the Pre-ISO and ISO Era (*p* < 0.001). The HCC characteristics of the transplanted cases are summarized in Table 3.

Comparing the LT patients in the Pre-ISO vs. ISO Era, the Pre-ISO patients were younger (median value 55 vs. 58 years; *p* = 0.02) and had a higher AFP level at diagnosis (9.8 vs. 6.1 ng/mL; *p* = 0.001).

As for the HCC characteristics, despite no differences in number (*p* = 0.07) and size of the major nodule (*p* = 0.37) bring found, a higher number of patients initially exceeding the Milan Criteria at WL inscription were finally transplanted in the ISO Era (25.3 vs. 15.5%; *p* = 0.048). Again, in both periods, a high percentage of patients received pre-LT neo-adjuvant treatment (94.2 vs. 90.8%; *p* = 0.30), and no significant differences in the type of LRT applied were found.

The MELD score at LT was different between the Pre-ISO Era and the ISO Era, with a higher laboratory MELD score in the former (14 vs. 12; *p* = 0.01). At the last imaging before LT, no difference between eras was found in number (*p* = 0.12) or maximum diameter (*p* = 0.57). However, in the ISO Era, more patients had a complete pathological response (cPR) (45.6 vs. 31.1%; *p* = 0.009) and a lower AFP level at LT (9 vs. 5 ng/mL; *p* = 0.001). In addition, the time between the last radiological imaging (Contrast-Enhanced Computed Tomography or Magnetic Resonance Imaging) and LT was significantly inferior in the ISO Era (3.0 vs. 2.1 months; *p* = 0.009).

This evidence was also confirmed in explant pathology, with a higher rate of cPR in the ISO Era (30.5% vs. 16.8%, *p* = 0.01).

### 3.4. Risk Factors for Post-LT Outcomes

Pepe and Mori’s test showed a higher but not statistically relevant cumulative incidence of LT cases in the ISO period when compared with the Pre-ISO period (*p* = 0.30) (Supplementary Figure S2). Comparing the different cumulative incidences in the Pre-ISO vs. ISO period, values of 13.2 (95%CI = 8.4–19.1) vs. 14.7 (95%CI = 10.7–19.3) at 3 months, 23.7 (95%CI = 17.3–30.7) vs. 29.1 (95%CI = 23.7–34.7) at 6 months, and 40.1 (95%CI = 32.3–47.8) vs. 50.8 (95%CI = 44.5–56.7) at 12 months were reported, respectively.

When the ISO system was explored regarding post-LT recurrence (Supplementary Figure S3) and overall survival (Supplementary Figure S4), no statistical differences were observed concerning the Pre-ISO Era.

**Figure 4:**
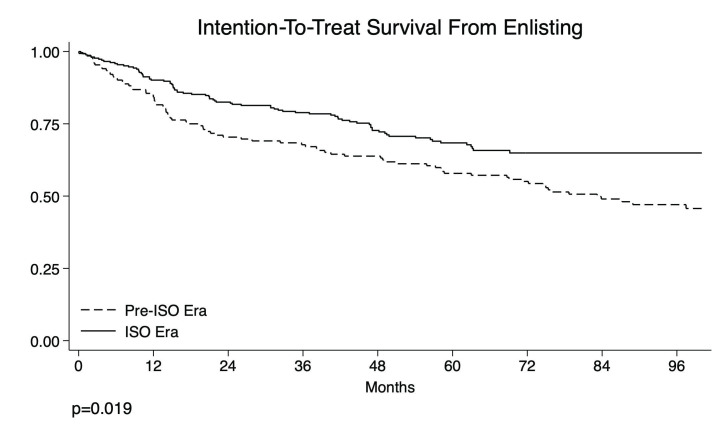
Intention-to-treat overall survival.

Conversely, in the ITT-OS analysis, patients transplanted in the ISO Era showed better rates at 1, 2, and 3 years compared with patients of the Pre-ISO Era (89%, 80%, and 74% vs. 79%, 67%, and 63%; *p* = 0.019) (Figure 4).

## 4. Discussion

The present study investigates the impact of a novel allocation policy for patients with HCC awaiting LT. According to this study’s findings, the adoption of the ISO reports promising results, with reduced Drop-Outs and intention-to-treat deaths and no increased risk of post-LT recurrence.

The ISO represents a further refinement of a process started over twenty years ago aimed at finding a preCIe regulation among HCC and non-HCC transplant candidates. Due to the relevant discrepancy among the number of donors and LT candidates, a fair allocation policy should be implemented to minimize the risk of Drop-Outs [20], avoiding disproportionate prioritization of HCC patients over non-HCC patients [21] and maintaining acceptable post-LT HCC recurrence rates [22].

In non-HCC candidates, the MELD score and its evolutions represent the best tools for prioritizing LT candidates. However, when these scores are used for prioritizing tumor patients, we risk violating the rule of “equity”, namely a specific subclass of patients is favored, harming the other one in terms of increased Drop-Out rates [23].

In the US, the typical strategy for overcoming this limitation has been to add MELD exception points in HCC individuals, with several modifications made during the years to reduce possible imbalances [24,25].

In recent years, more mathematically sophisticated “prioritization” systems have been attempted worldwide [21,26,27,28,29,30]. Among them, we can report the HCC-MELD proposed in 2006 [26], and its statistical refinement using a competing risks analysis [27], in which MELD and AFP values showed a relevant role in the setting of Drop-Out prediction. In 2012, a large study from the US proposed the Drop-Out-equivalent MELD (deMELD) score, a complex score based on several tumor- and patient-related covariates [28]. Another study reported the MELDEQ, in which the combination of the MELD score, AFP, tumor number, and tumor dimensions was reported [29]. Finally, a study from Cleveland proposed a continuous risk score integrating the MELD score and tumor-related variables to longitudinally assess the risk for Drop-Out and ITT death [30]. Unfortunately, these systems failed to be routinely adopted in national systems.

HCC represents the most frequent indication for LT in Italy [31]; therefore, mitigation of Drop-Out risk in this country is paramount. An allocation approach based on the “transplant benefit” instead of the “priority” has been proposed in this setting. The HCC-MELD score, based on the combination of AFP and the MELD score, has been developed to obtain a transplant benefit equal to that of non-HCC patients with the same numerical value of the MELD score [14]. HCC-MELD has been applied and incorporated into the ISO, in which a “blended” allocation approach has been applied based on the principles of urgency, utility, and transplant benefit [3].

The success of the ISO in the allocation process has already been evaluated in the setting of patients with advanced liver disease (MELD > 30) [32], but the present study is the first one exploring in detail the effect of the ISO on an HCC population.

As already reported, the positive effects of ISO application in our center resulted in a decrease in the rate of Drop-Outs, an improvement in the ITT-OS rates, and a rate of post-LT recurrence that was not improved. The mainstay of the ISO is the prioritization of patients concerning their evolution throughout oncological history. This patient prioritization does not rely merely on a snapshot of a patient’s oncological status at the time of listing or LT but on a dynamic process in which the morphological and biological aspects of the tumor are re-evaluated according to the response after LRT [23].

The impact of LRT has been explored in the settings of bridging and downstaging, showing how the response after treatment is relevant in terms of post-LT recurrence and Drop-Out during the WL period [33,34,35].

A study from the US reported that LT candidates with complete response or normal AFP after the first LRT had a negligible 1-year and 2-year risk of Drop-Out [33]. A large European study exploring the concept of ITT survival benefit reported that patients with complete response after LRT showed the lowest survival benefit with respect to the other classes of response [34].

This approach is present in the ISO, in which the patients with complete response after LRT do not receive any priority point (i.e., Stratum 3).

Conversely, early recurrence (<12 months) after LRT is based on the highest prioritization. This concept has also been well addressed in the literature, in which the necessity of multiple LRT treatments during the WL period due to the appearance of local recurrence or new nodules had a detrimental effect. A European study exploring the effect of multiple bridging treatments in patients meeting the Milan Criteria showed that the necessity to perform ≥ 4 LRTs was detrimental in terms of HCC-dependent LT failure (i.e., pre-transplant tumor-related delisting or post-transplant recurrence) [36].

The present study presents certain constraints. This study is retrospective and monocentric. The importance of validating these findings in other Italian centers employing the same allocation policy and, secondly, in an international setting are a relevant issue. Additionally, it is crucial to note that this analysis pertains to the only Italian region with a unified waiting list. Emilia-Romagna is unique in having two high-volume transplant centers that, unlike the rest of Italy, share a common waiting list, with rates of organ offerings and graft acceptance extremely high. Moreover, during the study period, the number of organ donor observations in the Emilia-Romagna region progressively increased, peaking at 270 in 2019 [10]. This expansion of the donor pool over the last decade contributed to a 20.6% increase in donor observations and a 22.2% increase in liver transplants for all indications during the ISO period compared to the Pre-ISO period. This improvement in organ availability likely played a key role in reducing the risk of Drop-Out among patients on the transplant waiting list.

Another critical aspect to underline is that the present study did not explore in detail the potential harm in adopting the ISO for non-HCC patients on the WL. This aspect was not a specific aim of the present analysis and requires further analyses.

## 5. Conclusions

In conclusion, the ISO policy showed a positive effect on Drop-Out decline without any detrimental effect in terms of post-LT recurrence, therefore suggesting a judicious selection of LT HCC candidates.

## Figures and Tables

**Table 1:** Clinical characteristics of enlisted patients.

** **	**Pre-ISO Era** **(n = 152)**	**ISO Era** **(n = 258)**	**Overall** **(N = 410)**	***p***
** **	**Median (IQR) or n (%)**			** **
Delisting Cause				
Liver transplantation	103 (67.8%)	217 (84.1%)	320 (78.0%)	<0.001
Drop-Out	49 (32.2%)	41 (15.9%)	90 (22.0%)	
Age at enlisting, years	57.1 (50.5–61.4)	57.5 (52.5–63.3)	57.4 (51.8–62.6)	0.04
Age at delisting, years	58.2 (51.8–62.8)	58.9 (53.2–64.6)	58.6 (52.7–63.7)	0.07
BMI	25.6 (23–27.8)	26.2 (24.1–29)	26 (23.9–28.6)	0.02
Sex, male	126 (82.9%)	218 (84.5%)	344 (83.9%)	0.67
HCV-related cirrhosis	84 (55.3%)	130 (50.4%)	214 (52.2%)	0.34
HBV-related cirrhosis	47 (30.9%)	64 (24.8%)	111 (27.1%)	0.18
Alcohol-related cirrhosis	23 (15.1%)	41 (15.9%)	64 (15.6%)	0.84
NASH-related cirrhosis	18 (11.8%)	35 (13.6%)	53 (12.9%)	0.62
Cryptogenic-related cirrhosis	2 (1.3%)	7 (2.7%)	9 (2.2%)	0.35
Other	4 (2.6%)	6 (2.4%)	10 (2.5%)	0.91
Blood group				0.20
0	53 (34.9%)	113 (43.8%)	166 (40.5%)	
A	71 (46.7%)	94 (36.4%)	165 (40.2%)	
B	19 (12.5%)	37 (14.3%)	56 (13.7%)	
AB	9 (5.9%)	14 (5.4%)	23 (5.6%)	
Previous surgery	96 (63.2%)	171 (66.3%)	267 (65.1%)	0.52
PVT				0.39
Absent	116 (76.3%)	201 (77.9%)	317 (77.3%)	
Partial	28 (18.4%)	37 (14.3%)	65 (15.9%)	
Complete	8 (5.3%)	20 (7.8%)	28 (6.8%)	
Diabetes	54 (35.5%)	64 (24.8%)	118 (28.8%)	0.02
Waiting list time, months	10.9 (4.6–19.9)	10.2(4.4–17.9)	10.6(4.4–18.1)	0.52

Abbreviations: HCV, hepatitis C virus; HBV, hepatitis B virus; NASH, nonalcoholic steatohepatitis; LRT, locoregional therapy; HCC, hepatocellular carcinoma; LRT, locoregional therapy; TIPS, transjugular intrahepatic porto-systemic shunt; MELD, model for end-stage liver disease; BMI, body mass index; LT, liver transplantation; AFP, alpha-fetoprotein; PVT, portal vein thrombosis.

**Table 2:** Hepatocellular carcinoma characteristics at enlisting.

** **	**Pre-ISO Era** **(n = 152)**	**ISO Era** **(n = 258)**	**Overall** **(N = 410)**	***p***
** **	**Median (IQR) or n (%)**			** **
Child–Pugh Score				
A	142 (39.0)	43 (29.3)	99 (45.6)	0.007
B	130 (35.7)	60 (40.8)	70 (32.3)	
C	92 (25.3)	44 (29.9)	48 (22.1)	
Number of HCC tumors at diagnosis				0.06
1	81 (54.4%)	110 (42.6%)	191 (46.9%)	
2	31 (20.8%)	78 (30.2%)	109 (26.8%)	
3	26 (17.4%)	41 (15.9%)	67 (16.5%)	
>3	11 (7.4%)	29 (11.2%)	40 (9.8%)	
Maximum HCC diameter at diagnosis				0.41
≤20 mm	59 (39.3%)	115 (44.6%)	174 (42.6%)	
20–50 mm	85 (56.7%)	129 (50.0%)	214 (52.5%)	
≥50 mm	6 (4.0%)	14 (5.4%)	20 (4.9%)	
Milan Out at diagnosis	32 (21.1%)	65 (25.2%)	97 (23.7%)	0.34
AFP diagnosis, ng/mL	7.0 (4.0–26.0)	6.1 (3.5–14.1)	6.8 (3.7–17)	0.35
LRT	141 (92.8%)	232 (89.9%)	373 (91.0%)	0.33
LR	34 (22.4%)	54 (20.9%)	88 (21.5%)	0.73
Ablation	77 (51.0%)	132 (52.0%)	209 (51.6%)	0.85
LR + ablation	97 (64.7%)	33 (44.6%)	130 (58.0%)	0.004
TACE	93 (61.6%)	135 (53.1%)	228 (56.3%)	0.10
Y-90	2 (1.3%)	14 (5.5%)	16 (4.0%)	0.04
Other treatments	2 (1.3%)	19 (7.3%)	21 (5.1%)	0.007

Abbreviations: AFP, alpha-fetoprotein. LRT, locoregional therapy; HCC, hepatocellular carcinoma; LR, Liver Resection; TACE, trans-arterial chemoembolization; Y-90, Yttrium-90 Radioembolization.

**Table 3:** HCC characteristics of transplanted patients.

**Variables**	**Pre-ISO Era (n = 103)**	**ISO Era** **(n = 217)**	**Overall** **(N = 320)**	***p***
** **	**Median (IQR) or n (%)**			** **
LRT	97 (94.2%)	197 (90.8%)	294 (91.9%)	0.3
LR	22 (21.4%)	47 (21.7%)	69 (21.6%)	0.95
Ablation	52 (50.5%)	113 (52.8%)	165 (52.1%)	0.70
LR + ablation	64 (62.7%)	11 (32.4%)	75 (55.1%)	0.002
TACE	66 (64.1%)	114 (53.3%)	180 (56.8%)	0.07
Y-90	2 (1.9%)	12 (5.6%)	14 (4.4%)	0.14
Other treatments	1 (1%)	14 (6.4%)	15 (4.7%)	0.031
Number of HCC tumors at diagnosis				0.07
1	60 (58.3%)	95 (43.8%)	155 (48.4%)	
2	22 (21.4%)	66 (30.4%)	88 (27.5%)	
3	16 (15.5%)	35 (16.1%)	51 (15.9%)	
>3	5 (4.9%)	21 (9.7%)	26 (8.1%)	
Maximum HCC diameter at diagnosis				0.37
≤20 mm	41 (39.8%)	99 (45.6%)	140 (43.8%)	
20–50 mm	58 (56.3%)	105 (48.4%)	163 (50.9%)	
≥50 mm	4 (3.9%)	13 (6.0%)	17 (5.3%)	
Milan Out at diagnosis	16 (15.5%)	55 (25.3%)	71 (22.2%)	0.048
AFP diagnosis, ng/mL	9.8 (4.0–30.0)	6.1 (3.8–15.0)	7.1 (4.0–19.0)	0.03
Last HCC number before LT				0.12
0	32 (31.1%)	101 (46.5%)	133 (41.6%)	
1	35 (34.0%)	57 (26.3%)	92 (28.7%)	
2	15 (14.6%)	28 (12.9%)	43 (13.4%)	
3	12 (11.7%)	17 (7.8%)	29 (9.1%)	
>3	9 (8.7%)	14 (6.5%)	23 (7.2%)	
Last maximum HCC diameter before LT				
≤20 mm	49 (69%)	88 (76%)	137 (73.3%)	0.57
20–50 mm	20 (28.2%)	26 (22.4%)	46 (24.6%)	
≥50 mm	2 (2.8%)	2 (1.7%)	4 (2.1%)	
AFP at LT, ng/mL	9.0 (4.0–31.0)	5.105 (3.0–12.1)	6.0 (3.1–18.2)	0.001
Radiological cRPR	32 (31.1%)	101 (46.5%)	133 (41.6%)	0.009
MELD pre-LT	14 (9–19)	12 (9–16)	12 (9–17)	0.01
Histological cPR	17 (16.8%)	65 (30.5%)	82 (26.1%)	0.01
Last imaging before LT, months	3.0 (1.3–5.4)	2.1 (1.2–3.8)	2.3 (1.2–4.4)	0.009
Waiting list time, months	8.7 (4.2–17.6)	9.2 (4.1–16.8)	9.02 (4.2–17.3)	0.81
Waiting list > 6 months	68 (66.0%)	142 (65.4%)	210 (65.6%)	0.92
Recurrence	15 (14.7%)	25 (11.5%)	40 (12.5%)	0.414

Abbreviations: AFP, alpha-fetoprotein; cPR, complete pathological response; HCC, hepatocellular carcinoma; LRT, locoregional therapy; LT, liver transplantation; LR, Liver Resection; MELD, model for end-stage liver disease; TACE, trans-arterial chemoembolization; Y-90, Yttrium-90 Radioembolization.

## Data Availability

The data supporting the findings of this study are available from the corresponding author (M.R.) on request according to national and international legislation regarding privacy and data protection.

## Supplementary Materials

The following supporting information can be downloaded at: https://www.mdpi.com/article/10.3390/cancers17101720/s1, Figure S1: Study Design; Figure S2: Cumulative Incidence of Liver Transplantation; Figure S3: Recurrence-Free Survival of Transplanted Patients; Figure S4: Overall Survival of Transplanted Patients.

## Abbreviations

ACLF	Acute On Chronic Liver Failure
AFP	alfa-fetoprotein
BCLC	Barcelona Clinic Liver Cancer Staging
BMI	body mass index
CI	confidence interval
cPR	complete pathologic response
ceCT	Contrast-Enhanced Computed Tomography
ceMRI	Magnetic Resonance Imaging
CTP	Child–Pugh Score
DCD	Donation After Cardiac Death
ECD	Extended Criteria Donor
FU	follow-up
HCC	hepatocellular carcinoma
HCV	hepatitis C virus
HBV	hepatitis B virus
IQR	interquartile range
ISO	Italian Score for Organ Allocation
ITT	intention-to-treat survival
LRT	locoregional treatment
LR	Liver Resection
LT	liver transplantation
MELD	model for end-stage liver disease
mVI	microvascular Invasion
MWA	microwave ablation
NASH	nonalcoholic steatohepatitis
OR	odds ratio
OS	overall survival
PEI	Percutaneous Ethanol Injection
PVT	portal vein thrombosis
RECIST	Response Evaluation Criteria In Solid Tumors
RER	Regione Emilia-Romagna
RFA	radiofrequency ablation
RFS	recurrence-free survival
sHR	sub-hazard ratio
SE	Standard Error
SIRT	Emilia-Romagna Donor Manager Software
TACE	trans-arterial chemoembolization
TIPS	transjugular intrahepatic porto-systemic shunt
ULD	underlying liver disease
WL	waiting list
Y-90	Yttrium-90 radioembolization
