# Supplementary material for: The Italian Score for Organ Allocation: A Ten-Year Monocentric Retrospective Analysis in Liver Transplantation for Hepatocellular Carcinoma
Source: Cancers (Basel). 2025 May 21;17(10):1720. doi: 10.3390/cancers17101720 (PMC12110210; doi:10.3390/cancers17101720)

Supplementary Material

Supplementary Figure S1. Study Design

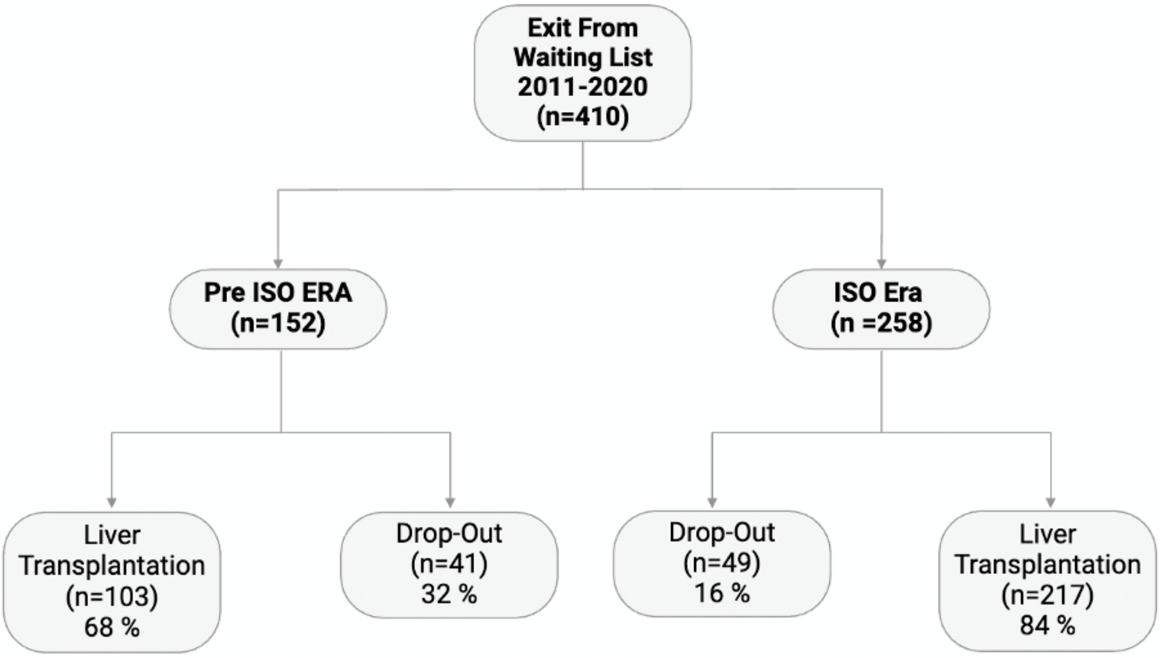

Supplementary Figure S2. Cumulative Incidence of Liver Transplantation

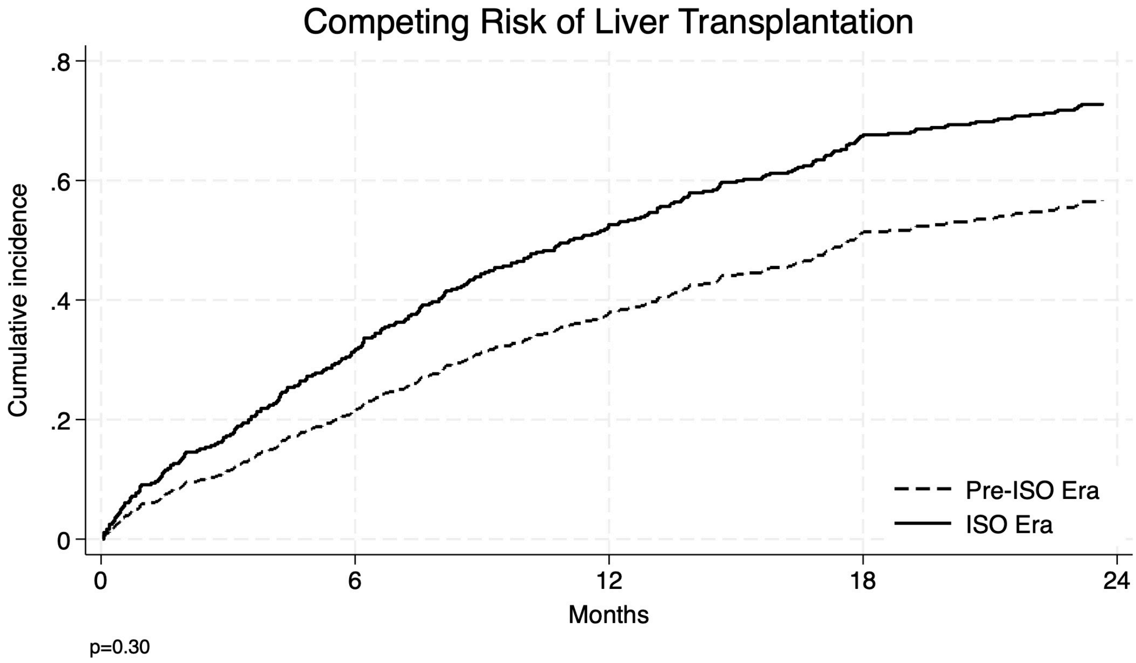

Supplementary Figure S3. Recurrence-Free Survival of Transplanted Patients

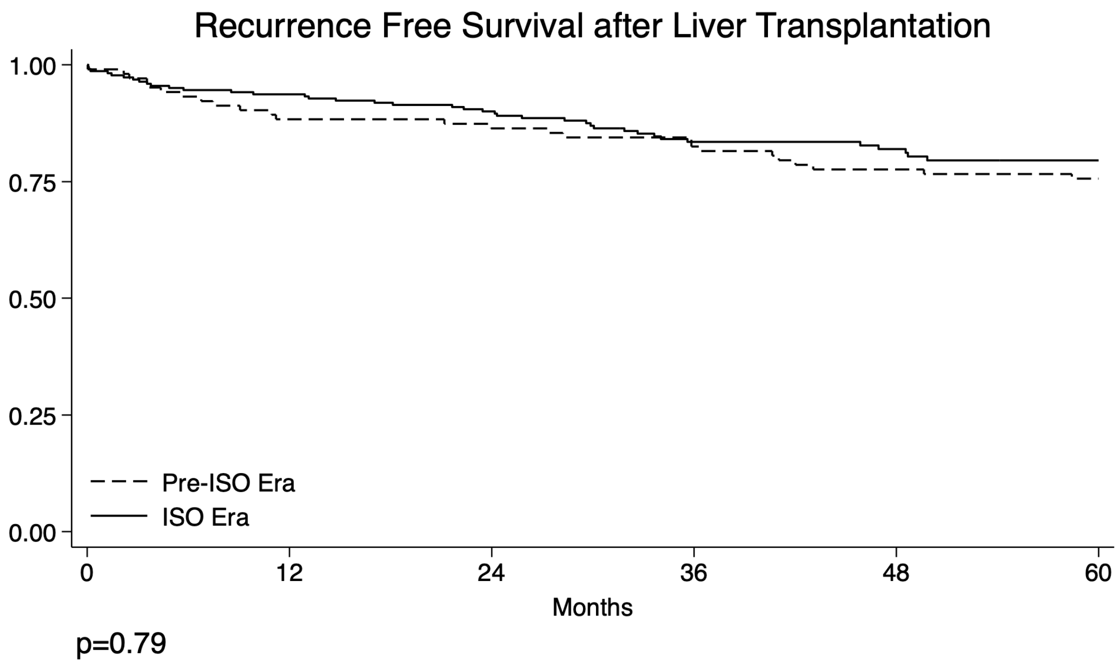

Supplementary Figure S4. Overall Survival of Transplanted Patients

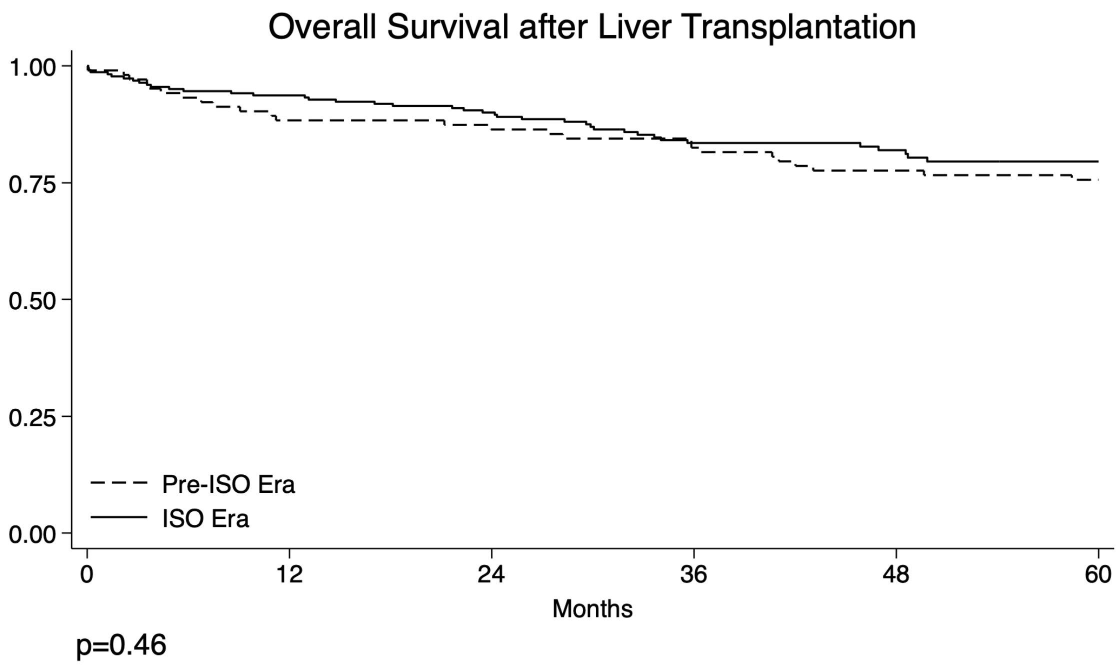

Supplement: Supplementary file 1 [file cancers-17-01720-s001.zip › cancers-3607703-supplementary.pdf]
